# Results of Endoscopic Dacryocystorhinostomy under Local Anesthesia with Minimal Sedation

**DOI:** 10.1155/2017/6712491

**Published:** 2017-07-16

**Authors:** Woong Chul Choi, Ji-Sun Paik, Sang Hee Doh

**Affiliations:** ^1^Myoung Eye Plastic Surgery Clinic, SH Tower 3rd Floor, Gangnam Gu, Nonhyeon Dong, Seoul 278-22, Republic of Korea; ^2^Department of Ophthalmology and Visual Science, Seoul St. Mary's Hospital, College of Medicine, The Catholic University of Korea, Seoul, Republic of Korea; ^3^Department of Ophthalmology and Visual Science, St. Paul's Hospital, College of Medicine, The Catholic University of Korea, Seoul, Republic of Korea

## Abstract

**Purpose:**

We evaluated the tolerability and efficacy of endoscopic dacryocystorhinostomy (Endo-DCR) in patients treated in the leaning position and under local anesthesia with minimal sedation (LAS).

**Study Design:**

Questionnaire to determine subjective success of Endo-DCR.

**Methods:**

From May 2013 to August 2014, a total of 95 eyes with epiphora presented to the Myoung Eye Plastic Surgery Clinic in Seoul, Korea, and were treated with Endo-DCR under LAS. Three nerve blocks were administered to achieve local anesthesia. Postoperatively, the wound site was packed with Nasopore to control bleeding and promote wound healing. Outcome measures included a patient questionnaire completed on postoperative day 7 to evaluate intraoperative and postoperative pain based on the VAS (0 to 10).

**Results:**

Mean intraoperative and postoperative pain scores were 1.03 and 1.64, respectively, for 95 eyes. Of the 95 eyes treated, the patients in 82 eyes (86.31%) reported that they would prefer LAS over GA for a repeat Endo-DCR. The subjective and objective surgical success rates were 90.14% and 95.77%, respectively.

**Conclusions:**

Endo-DCR carried out under LAS with the patient in the leaning position is more useful, efficient, and feasible than Endo-DCR performed under GA with the patient in the supine position.

## 1. Introduction

Since external dacryocystorhinostomy (Ext-DCR) was first described in 1904 by Toti [1], various techniques for endoscopic dacryocystorhinostomy (Endo-DCR) have been developed, and overall success rates for both procedures have been reported to be as high as 95% [2].

For the DCR procedure, most surgeons prefer general anesthesia (GA) to local anesthesia (LA), because GA can completely eliminate pain during periosteal elevation and the creation of an osteotomy [3]. However, GA may be extremely hazardous in elderly patients and may be associated with extensive systemic and metabolic disorders [4]. Because GA can pose many problems, such as intraoperative bleeding and postoperative epistaxis [5], various LA techniques have been introduced by Hurwitz et al. [6], Fanning [7], Smith et al. [8], and Maheshwari [9]. Such techniques not only reduce the risks associated with GA (e.g., postoperative nausea and vomiting) but also enable shorter operation times and faster recovery.

We evaluated the tolerability and efficacy of Endo-DCR performed with the patient in the sitting position under local anesthesia with sedation (LAS). The visual analogue scale (VAS) was used to determine patients' perceptions of pain, a postoperative questionnaire was used to evaluate subjective success, and the nasolacrimal syringing test was employed to determine objective improvements.

## 2. Methods

### 2.1. Patients

From May 2012 to August 2014, a total of 92 patients (95 eyes) presented with epiphora to the Myoung Eye Plastic Surgery Clinic in Seoul, Korea, and were treated with Endo-DCR under LAS. The mean age of the patients was 58.32 years (range, 31–80), all of whom provided informed consent for data analysis. This study was approved by the institutional review board and was conducted in accordance with the Declaration of Helsinki.

### 2.2. Surgical Procedure

**Figure 1:**
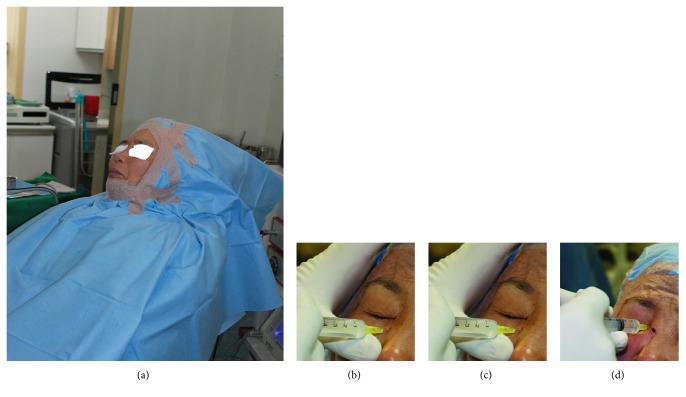
(a) Preparation of patient in sitting position. (b) Infraorbital nerve block. (c) Medial canthal nerve block. (d) Ethmoidal nerve block.

Bosmin gauze (1 : 100,000 epinephrine) (Jeil Pharmaceutical, Seoul, Korea) was used to pack the middle turbinate for 10 minutes before the patients were assisted to the leaning position without the chin up to minimize the posterior nasal influx of blood, which irritates the airway (trachea or epiglottis), and to facilitate the intraoperative removal of posterior nasal influx material by oral suction (Figure 1(a)). Local anesthetic was administered with a 30-gauge needle at three points to achieve medial canthal, infraorbital, and ethmoidal nerve blocks (Figures 1(b), 1(c), and 1(d)), and a total of 5.5 mL of 2% lidocaine mixed with 1 : 100,000 epinephrine was injected at these three sites.

**Figure 2:**
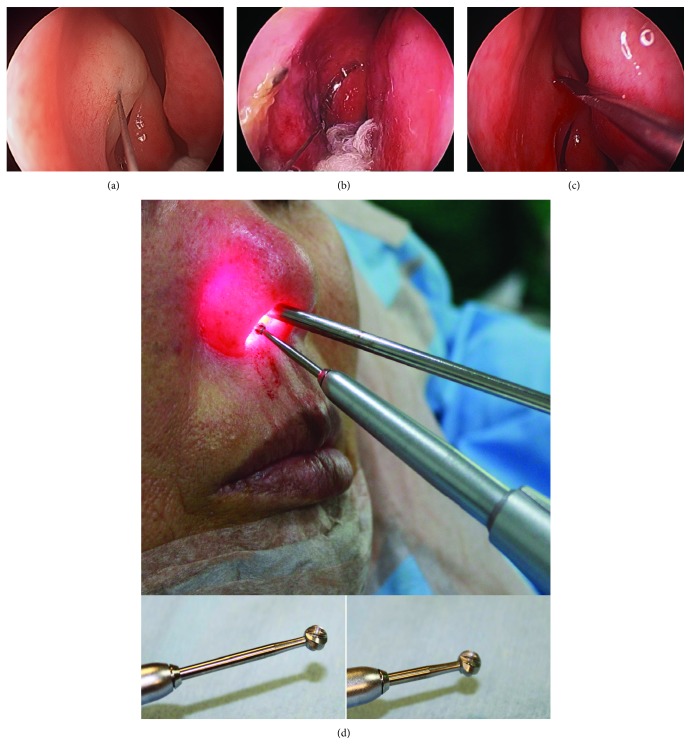
(a) Administering anesthetic to nasal mucosal flap. (b) Posterior nasal packing. (c) Incision site. (d) Adjustment of drill burr length (lower panels) and position of drill at operative site (upper panel).

A 4 mm diameter, rigid nasal endoscope was inserted into the nose (at a 0-degree angle), and an additional 1.5 mL of local anesthetic was injected into the area of the nasal mucosal flap around the axilla of the middle turbinate (Figure 2(a)). Posterior nasal packing was placed from the inferior meatus to the posterior middle turbinate for occlusion to minimize intraoperative retrograde bleeding into the posterior nasal cavity (Figure 2(b)). For conscious sedation, 2 to 4 mL of midazolam (1 mg/1 mL) was injected intravenously, with an induction time of 1 to 2 minutes (half-life = 1 hr), and pulse oximetry was used to monitor the oxygen concentration in the blood. The dose of midazolam depended on the patient's age and general condition. Initially, 2 mg (2 mL) of midazolam was intravenously injected over 2 to 3 minutes, and its sedative effect was evaluated after 2 minutes. An additional dose of 1 mg (1 mL) was given if the level of sedation was not sufficient; however, the total dose given did not exceed 4 mg because of the associated risk of respiratory depression.

A sickle knife was used to make a curvilinear incision in the mucosa starting at about 5 to 6 mm above the axilla of the middle turbinate (Figure 2(c)). This incision was brought inferiorly toward the insertion of the inferior turbinate. A Freer elevator was used to raise up the nasal mucosa to expose the axillary ridge (part of the frontal process of the maxillary bone) anterior to the lacrimal bone, and the tissue was resected using cutting forceps. An angled electric cutting drill (tip size, 3–5 mm) was used to make a bony ostium with a cutting speed ranging from 10,000 to 15,000 rpm. The length of the drill burr was adjusted according to the patient's nasal anatomy (Figure 2(d)), because an overlong burr often makes contact with the internal nostril during its rotation, making it difficult to avoid damage to surrounding nasal structures, including the nasal septum and lateral nasal wall. In patients with septal deviation, turbinate hypertrophy, or a narrow nostril, the length of the 3 mm drilling burr shaft was shortened to avoid mucosal injury, which could result in mucosal adhesion and could decrease the success rate of the Endo-DCR.

**Figure 3:**
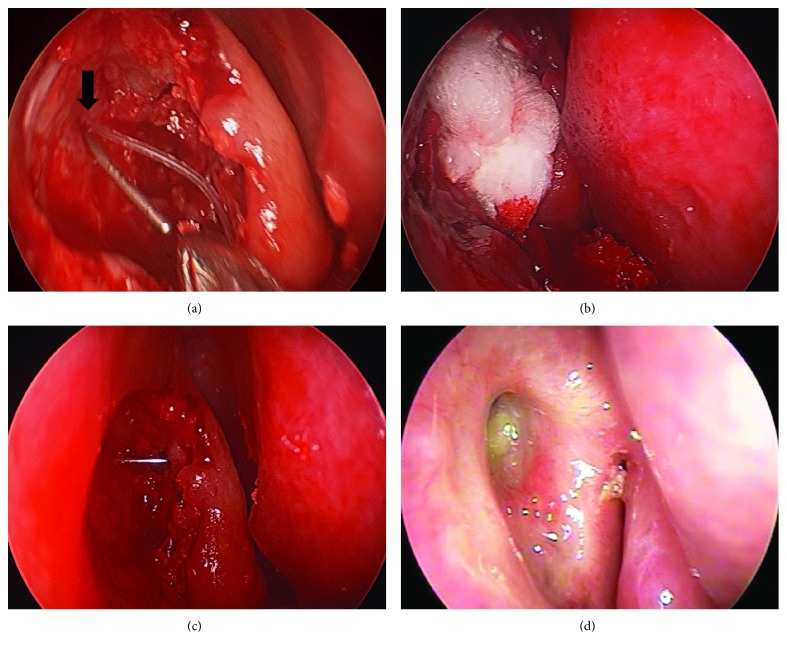
(a) Position of lacrimal tubes through the same internal opening of the common canaliculus. (b) Packing with Nasopore. (c) Horizontal probe passing through the lower canalicular plane. (d) At 6 months postoperatively, the internal opening is well formed.

After removing enough maxillary bone around the axilla of the middle turbinate (at least 1 cm in diameter), the lacrimal sac was tented up with a number 1 Bowman probe and was cut superiorly and inferiorly with a sickle knife to identify the opening of the common canaliculus. The anterior flap of the lacrimal sac was folded over in apposition to the nasal mucosa, and the posterior flap remnant was partially cut. If an uncinate process that interfered with tear flow was present, it was partially removed. The lacrimal tubes were passed through the same internal opening of common canaliculus from the upper and lower canaliculi and were retrieved endoscopically (Figure 3(a)). A piece of Nasopore (polyether ester urethane; Polyganics, Groningen, The Netherlands) was used for packing to stop the bleeding and improve wound healing (Figure 3(b)).

Patients were instructed to restrain from excessive physical activity, sleep with their heads elevated, and avoid nose blowing for 3 days after surgery to minimize nasal bleeding complications. Levofloxacin eye drops (Cravit, Santen, Japan), a topical antibiotic, and fluorometholone 0.1% eye drops (Taejoon Pharm, Seoul, Korea), a topical steroid, were applied four times a day for 2 months until extubation, and postoperative pain was controlled with an oral analgesic for 5 days.

**Figure 4:**
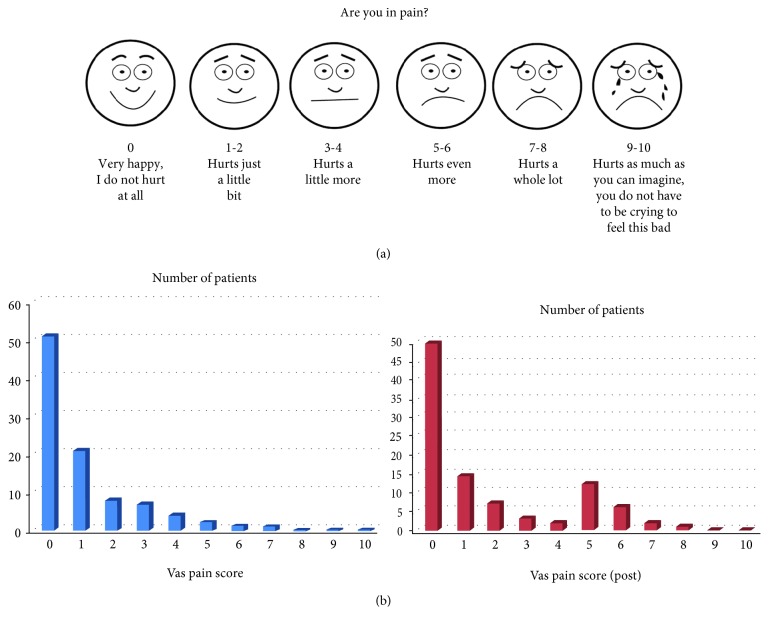
(a) Visual analogue scale (VAS) used by patients for subjective assessment of pain. (b) Distribution of intraoperative (blue) and postoperative (red) pain scores.

Intraoperative and postoperative pain scores were evaluated using a patient questionnaire on postoperative day 7 at the outpatient clinic. Patients were asked to use the six-level VAS to report their degree of pain based on a score of 0 to 10, with 0 indicating no pain and 10 indicating the greatest pain (Figure 4(a)). In addition, the patients were asked whether they would undergo the Endo-DCR under LAS again if given the option of GA for treatment of the other eye.

### 2.3. Statistical Analysis

A paired *t*-test was used to assess the intraoperative and postoperative pain scores with the use of SPSS software version 20 (IBM, New York, United States).

## 3. Results

From May 2013 to August 2014, there were 92 patients (95 eyes) with epiphora treated with Endo-DCR under LAS at the Myoung Eye Plastic Surgery Clinic. Of these 92 patients, 78 (80 eyes) were women (84.78%). The patients ranged in age from 31 to 80 years, with a mean (±SD) age of 58.32 ± 10.65 years. Fifty percent (46 of 92) of patients were older than 60 years of age; systemic diseases in this older group included high blood pressure (18), diabetes mellitus (6), hyperlipidemia (5), renal function disorder (3), and liver disorder (3), all of which can be risk factors when GA is used. In addition, 10 of the patients over age 60 took aspirin for anticoagulation.

Among the 95 eyes treated, the two main causes of epiphora were chronic dacryocystitis (45 eyes [47.37%]) and primary acquired nasolacrimal duct obstruction (34 eyes [35.80%]). Other causes included nasolacrimal duct stenosis (9 eyes [9.47%]), lower canalicular obstruction (3 eyes [3.16%]), functional nasolacrimal duct obstruction (35 eyes [3.16%]), and common canalicular obstruction (1 eye [1.05%]). The use of local anesthesia at three sites during the operation successfully controlled pain, and the administration of intravenous midazolam did not interfere with patients' ability to expectorate posterior nasal blood.

Intraoperative and postoperative pain scores as assessed by patients using the VAS (Figure 4(a)) are shown in Table 1 and Figure 4(b). For the intraoperative results, 91 out of 95 eyes (95.79%) reported pain scores under 4 (hurts a little more), with a mean (±SD) score of 1.03 ± 1.53 (hurts just a little bit). For the postoperative results, 74 out of 95 eyes (77.89%) reported pain scores under 4 (hurts a little more), with a mean (±SD) score of 1.64 ± 2.21 (hurts just a little bit). The mean intraoperative pain score was significantly lower than the mean postoperative pain score on a paired *t*-test (*p* value = 0.015).

When offered the option of undergoing Endo-DCR under GA if their other eye needed to be treated, 76 of the 92 patients (82.61%) said they would prefer Endo-DCR under LAS again, even though 22.11% of the patients reported a postoperative pain score above 5 (Figure 4(b)). In 3 of the 76 patients, both eyes were treated using the same procedure (Endo-DCR under LAS).

The average duration of the Endo-DCR was 30 minutes. In cases involving patients who took anticoagulant therapy or had narrow nasal cavities, the operation took longer. Posterior nasal bleeding during surgery was effectively controlled by oral suction. For the 71 eyes followed up for more than 6 months, the rate of subjective surgical success was 90.14% (64/71) and the rate of objective surgical success was 95.77% (68/71). The three recurrent cases were caused by granuloma, with lower canalicular obstruction in two. In terms of postoperative complications, nasal bleeding was the most common (53/95 eyes [55.79%]), but it subsided within a week after the operation and did not require any specific coagulative treatment. Other minor complications, including nasal stuffiness and headache, also subsided within a week.

## 4. Discussion

Significant intraoperative bleeding during Endo-DCR under GA is a well-known complication that can prolong surgery and frequently results in postoperative epistaxis, because GA promotes venous engorgement and vasodilation [10]. Furthermore, Endo-DCR under GA in patients at high risk may be life threatening, necessitating the presence of a highly skilled anesthesia team at a high cost, and can lead to hospital admission, requiring the availability of an intensive care unit to manage severe complications. When Endo-DCR is performed under local anesthesia with the patient in the supine position, intraoperative bleeding can cause discomfort owing to the posterior nasal influx of blood and irrigation fluid, which can result in aspiration. In contrast, by operating with the patient in the leaning position and using posterior nasal packing, we were able to effectively control posterior nasal influx. Intraoperative posterior nasal influx irritates the airway and stimulates the coughing reflex. In such cases, airway obstruction was prevented by promptly positioning the patient so that the chin was aimed toward the chest, allowing the blood to be easily removed by oral suction or flow down spontaneously. These are the advantages of performing Endo-DCR with the patient in the leaning, as opposed to the supine, position. The clear demonstration of the anatomical landmarks can be possible using a 0-degree rigid endoscope (Figure 2(d)), and for creating the bony ostium, enough maxillary bone must be removed to ensure that the horizontal probe can pass through to the lower canalicular plane (Figure 3(c)) to allow a common canalicular opening into the nasal cavity for better postoperative surgical success (Figure 3(d)).

While the visual analogue pain score may suffice to quantify pain, the amnesic effect of midazolam can result in the mean intraoperative pain score being lower than the mean postoperative pain score; however, it is more important to have the patient consciously sedated to allow the operation to go more smoothly. Furthermore, the average score for patients' recollection of intraoperative pain was 1.03 (hurts just a little bit), allowing patients to accept Endo-DCR under LAS again instead of the same procedure performed under the more complicated GA.

In our study, two patients (4 eyes) reported intraoperative pain scores on VAS of between 5 (hurts even more) and 7 (hurts a whole a lot), but they chose Endo-DCR under LAS again for their other eye. The other possible reasons why 82.61% of the patients said they would prefer Endo-DCR in the sitting position under LAS rather than under GA are the less complicated preoperative preparation and the psychological benefit of being stabilized and able to return home after only 30 minutes in the recovery room.

## 5. Conclusions

In conclusion, Endo-DCR in the leaning position under LAS can be used as a useful, rapid, and more feasible alternative to Endo-DCR in the supine position under either GA or LA, especially when GA would pose some risk or for patients who do not want GA.

## Figures and Tables

**Table 1:** Intraoperative versus postoperative pain scores, as reported by 92 patients (95 eyes) using the visual analogue scale (VAS).

VAS pain score	Intraoperative	Postoperative
	Number of eyes	
0 (no pain)	51	48
1	21	14
2	8	7
3	7	3
4	4	2
5	2	12
6	1	6
7	1	2
8	0	1
9	0	0
10 (greatest pain)	0	0
Total eyes	95	95
